# Top-down control of saccades requires inhibition of suddenly appearing stimuli

**DOI:** 10.3758/s13414-020-02101-3

**Published:** 2020-08-16

**Authors:** Christian Wolf, Markus Lappe

**Affiliations:** Institute for Psychology, University of Muenster, Fliednerstrasse 21, 48149 Münster, Germany

**Keywords:** Eye Movements: Mechanisms, Eye movements and visual attention

## Abstract

Humans scan their visual environment using saccade eye movements. Where we look is influenced by bottom-up salience and top-down factors, like value. For reactive saccades in response to suddenly appearing stimuli, it has been shown that short-latency saccades are biased towards salience, and that top-down control increases with increasing latency. Here, we show, in a series of six experiments, that this transition towards top-down control is not determined by the time it takes to integrate value information into the saccade plan, but by the time it takes to inhibit suddenly appearing salient stimuli. Participants made consecutive saccades to three fixation crosses and a vertical bar consisting of a high-salient and a rewarded low-salient region. Endpoints on the bar were biased towards salience whenever it appeared or reappeared shortly before the last saccade was initiated. This was also true when the eye movement was already planned. When the location of the suddenly appearing salient region was predictable, saccades were aimed in the opposite direction to nullify this sudden onset effect. Successfully inhibiting salience, however, could only be achieved by previewing the target. These findings highlight the importance of inhibition for top-down eye-movement control.

**Electronic supplementary material:**

The online version of this article (10.3758/s13414-020-02101-3) contains supplementary material, which is available to authorized users.

What we see is highly influenced by where we look. Where we direct our gaze and/or our attention can be stimulus driven or driven by behavioral goals and learned reward contingencies (for reviews, see Awh, Belopolsky, & Theeuwes, 2012; Corbetta & Shulman, 2002). Salient stimuli that suddenly appear in the periphery can cause an orienting response (Posner, 1980) and are particularly successful in capturing gaze and visual attention (Enns, Austen, Di Lollo, Rauschenberger, & Yantis, 2001; Ludwig & Gilchrist, 2002; Yantis & Jonides, 1984). This is also reflected in the firing rate of neurons in the lateral intraparietal area (LIP; Gottlieb, Kusunoki, & Goldberg, 1998; Kusunoki, Gottlieb, & Goldberg, 2000) and frontal eye fields (FEF; Joiner, Cavanaugh, Wurtz, & Cumming, 2017), areas which are said to combine information about salience and relevance, thus acting as a priority map of visual selection (Bisley & Mirpour, 2019; Gottlieb et al., 1998; Ptak, 2012; Thompson & Bichot, 2005). For example, neurons in LIP respond more strongly if a sudden onset stimulus is brought into their receptive field compared with a continuously displayed stimulus (Gottlieb et al., 1998), and neurons in FEF respond more strongly to stimuli flashed in temporal isolation compared with stimuli flashed in close temporal proximity (Joiner et al., 2017).

Distractors presented along with the designated target can also influence saccade target selection. In these cases, eye movements land at intermediate locations, a phenomenon referred to as *global effect* (Findlay, 1982; for reviews, see Van der Stigchel & Nijboer, 2011; Vitu, 2008). The global effect can be observed when target and distractor are presented in close spatial proximity (Walker, Deubel, Schneider, & Findlay, 1997). Actual endpoints depend on the relative salience of target and distractor (Deubel, Wolf, & Hauske, 1984) and on saccade latency: Early saccades are biased by the presence of the distractor to intermediate locations, whereas long-latency saccades are accurate (Coëffé & O’Regan, 1987; McSorley & Findlay, 2003; Ottes, Van Gisbergen, & Eggermont, 1985). The fact that the contribution of salience to saccade target selection strongly depends on the timing of the saccade is also reflected in a finding by Donk and van Zoest (2008): In an array of vertical (or horizontal) lines, two lines deviated from the cardinal axis. One of these two singletons was defined by a small the other by a large orientation contrast. Participants had to make an eye movement to the more salient singleton as defined by the orientation contrast. The proportion of correct selection was highest for short-latency saccades and decreased with latency, highlighting that salience has the strongest impact on early responses.

When salience and behavioral goals compete for oculomotor control, early saccades are biased towards salience, and long-latency saccades are biased towards behavioral goals (Ghahghaei & Verghese, 2015; Ludwig & Gilchrist, 2002; Salinas et al., 2019; Schütz, Trommershäuser, & Gegenfurtner, 2012; van Zoest, Donk, & Theeuwes, 2004). Schütz et al. (2012) measured saccade endpoints to a peripherally appearing stimulus consisting of a salient and a nonsalient but rewarded region. Early saccades were biased towards salience, and the strength of this bias depended on the physical salience of the target. With increasing latency, saccade endpoints showed a dynamic transition from the salient towards the rewarded region. This transition from bottom-up to top-down eye-movement control was interpreted in terms of the time it takes to integrate information about value into the saccade plan (Ghahghaei & Verghese, 2015; Schütz et al., 2012). However, at the same time that value information is integrated, an orienting response to the salient region must be inhibited. Therefore, the same transition would be expected if the time course is determined by the time it takes to successfully inhibit a response towards the salient region.

The aim of the present work is to dissociate these two possibilities and reveal whether the transition from bottom-up to top-down eye-movement control is determined by deliberate planning and integration of top-down information or by inhibiting responses to salient stimuli. The first three experiments (Experiment 1: sudden onset, Experiment 2: continuous display, Experiment 3: cued onset) directly focus on this question. In two further experiments, we investigate how responses to suddenly appearing salient stimuli can be successfully inhibited—first, whether inhibition can be achieved when the onset of a salient target is fully predictable (Experiment 4: predictable onset) and, second, whether successful inhibition depends on the quality with which a peripheral target can be previewed (Experiment 5: preview quality). In the last experiment (Experiment 6: blank onset), we test whether the reappearance of a salient stimulus can bias saccade endpoints, although it had been previewed beforehand.

## Experiments 1–3: The transition to top-down control requires inhibition

**Fig. 1:**
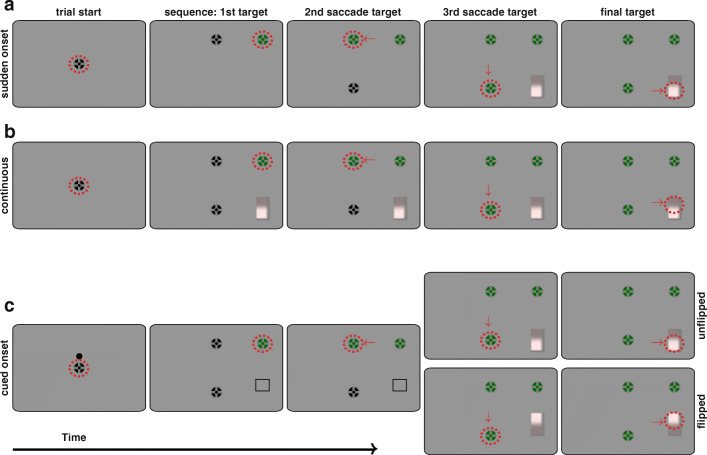
Trial procedure for Experiment 1 (sudden onset; **a**), Experiment 2 (continuous display; **b**) Experiment 3 (cued onset; **c**). Participants started trials by fixating a central cross and pressing the space bar (first column). Then, participants made saccades to a sequence of four targets: three fixation crosses and one vertical luminance bar. The luminance bar consisted of two regions: a high-salient region that was brighter than the background, and a low-salient region slightly darker than the background. In conditions with reward, participants received a reward when they managed to look at the low-salient region. Whereas the three fixation crosses in the sequence were always at the same location, the luminance bar could appear left or right from the last fixation cross with the rewarded region being either up or down. Red dashed circles and red arrows denote the current gaze location and saccades and were not displayed during the experiment. **a** In Experiment 1 (sudden onset), the next target was only displayed once the previous target was foveated. **b** In Experiment 2 (continuous display), all sequence targets were shown once participants fixated the upper right fixation cross. **c** In Experiment 3 (cued onset), participants knew the location of the rewarded region as soon as the sequence targets appeared (second column) by means of two cues, but they did not know the location of the salient region, which could appear above or below. A first cue was shown when participants initiated a trial (first column) and signaled whether the rewarded region will be up (dot above cross) or down (dot below cross) relative to the vertical midline of the last fixation cross. The second cue (columns two and three) was the outline of the rewarded region. The outline cue ensured that a saccade into the low-salient region could be planned before the luminance bar appeared. The first cue was added to additionally ensure that performance was not limited by peripheral location discrimination performance. The luminance bar could either appear vertically centered with the last fixation cross (unflipped) or the high-salient region appeared on other side of the low-salient region (flipped). (Color figure online)

In all experiments, participants had to make a sequence of four saccades. The first three targets were fixation crosses, whereas the last target was a vertical luminance bar consisting of a high-salient and a low-salient region (see Fig. 1). The dependent variable in all experiments was the vertical saccade endpoint on that luminance bar. Whereas the locations of the three fixation crosses were the same for every trial in every experiment, the luminance bar could appear to the left or right of the last fixation cross. The high-salient region could be in the upper or in the lower half of the luminance bar. Saccades into the low-salient region were rewarded in selected conditions.

The planning hypothesis would predict that successful top-down control of eye movements depends on the time given to plan a saccade, whereas the inhibition hypothesis would state that successful oculomotor control depends on the temporal difference between stimulus onset and response and thus on how long the salient and to-be-inhibited region was previewed. Because multiple saccades can be planned in parallel (McPeek, Skavenski, & Nakayama, 2000; McSorley, Gilchrist, & McCloy, 2020; McSorley, McCloy, & Williams, 2016; Quaia, Joiner, FitzGibbon, Optican, & Smith, 2010), using saccade sequences allowed us to independently manipulate the time given to saccade planning as well as the temporal onset of the vertical bar and thus the onset of the to-be-inhibited salient region. We used saccade sequences rather than a single saccade with a timed go cue, because presentation of such a go cue would have either required another visual onset or an event in another modality (e.g., auditory cue), either of which might confound saccade behavior (e.g., Vidal, Desantis, & Madelain, 2020).

In Experiment 1 (sudden onset), every saccade target (i.e., fixation crosses and luminance bar) only appeared after the previous target had been fixated (see Fig. 1a). The aim of this first experiment was to replicate the dynamic transition from bottom-up to top-down saccade control (Schütz et al., 2012) using saccade sequences instead of single reactive saccades. Thus, we expected short-latency saccades to be biased towards salience, whereas long-latency saccades should be directed towards the low-salient region when this region is associated with a reward.

In Experiment 2 (continuous display), all targets were continuously displayed throughout the trial once the first saccade target was fixated (see Fig. 1b). The aim of this experiment was to show that the transition to top-down control does not necessarily depend on whether participants initiate a quick or a slow oculomotor response (thus, whether the fixation duration on the last fixation cross is short or long), but that it depends on how long the target can be previewed. If this were the case, we would expect saccade endpoints to be directed towards the low-salient region when this region is associated with a reward. This should be true for all fixation durations.

In Experiment 3 (cued onset), all fixation crosses and the outline of the low-salient region (a black frame) were shown throughout the trial once participants fixated the first saccade target (see Fig. 1c). The luminance bar itself only appeared after fixating the last fixation cross, and the high-salient region could appear above or below the low-salient region. Thus, in rewarded trials of Experiment 3, participants knew throughout the trial where they want to saccade to, and had sufficient time to integrate that information into the saccade plan. However, they did not know the location of the high-salient region until the luminance bar appeared. We expected that saccades initiated shortly after the onset of the luminance bar would be biased towards salience, whereas late saccades would be successfully directed towards the low-salient region (inhibition hypothesis). In contrast to that, the planning hypothesis would predict that in conditions with reward, endpoints should be directed towards the low-salient region—independent of the temporal relationship between saccade onset and target onset.

### Methods

#### Participants

Participants were undergraduate students of the University of Muenster and were reimbursed with 8€/h or course credit. Additionally, participants could receive a reward depending on their individual performance. Every trial with reward could provide nine score points, and 100 score points were converted in 0.1€ at the end of the experiment. Twenty-four individuals participated in the cued onset experiment (mean age = 24 years, age range: 18–30 years, 20 females), of which 12 (mean age = 25, age range: 22–30 years, three males) also participated in the sudden onset and continuous display experiment. Participants of all experiments provided written informed consent before testing. Experiments were approved by the ethics committee of the Department of Psychology and Sport Sciences of the University of Muenster (Proposal No. 2018-18-ChW) and were conducted in accordance with the declaration of Helsinki.

#### Setup and stimuli

Stimuli were presented on an Eizo FlexScan 22-inch CRT monitor (Eizo, Hakusan, Japan) with a resolution of 1,152 × 870 pixels, a refresh rate of 75 Hz, and an effective display size of 40.7 × 30.5 cm viewed from 67 cm distance. Stimulus presentation was controlled via the Psychtoolbox (Brainard, 1997; Kleiner, Brainard, & Pelli, 2007) in MATLAB (The MathWorks, Natick, MA, USA). Eye position of the right eye was recorded at 1000 Hz using the EyeLink 1000 (SR Research, Ontario, Canada) and the EyeLink Toolbox (Cornelissen, Peters, & Palmer, 2002).

All stimuli were presented on a uniform gray background. In every saccade sequence, all targets but the last were fixation crosses consisting of a combination of bull’s eye and cross hair (Thaler, Schütz, Goodale, & Gegenfurtner, 2013) with a diameter of 0.5°. In all experiments, fixation crosses turned from black to green once they had been looked at. The final target was a vertical bar covering 1° in width and 3° in height. The bar consisted of two vertical parts of equal size: a high-salient and a low-salient region. The high-salient region was clearly brighter than the background, whereas the low-salient region was only slightly darker than the background (see Fig. 1). In conditions with reward, participants received a reward for saccades to the low-salient region. The transition from target to background and from high-salient to low-salient region was smoothed by means of a cumulative Gaussian with a standard deviation of 1/12° to prevent a sharp edge contrast.

#### Design and procedure

##### Experiment 1 (sudden onset)

Experiment 1 consisted of two conditions recorded in separate blocks: one condition with and one condition without reward. In the condition with reward, participants received a reward for a saccade to the low-salient region. Both blocks contained 200 trials and were recorded in different sessions. To prevent a transfer of learned reward relationships (e.g., Anderson, Laurent, & Yantis, 2011) or a transfer of top-down strategies to the unrewarded condition, the unrewarded condition was always recorded in the first session. It was recorded together with the unrewarded conditions of Experiments 2 and 3. In the second session, every participant completed the rewarded conditions of the three experiments in three different blocks. The order in which blocks of the three experiments were recorded was balanced across participants but consistent for the two sessions of every individual.

Every trial of both respective sudden onset conditions required participants to make four saccades (see Fig. 1a). Participants were instructed to successively look at the three fixation crosses and the luminance bar in the prescribed order. They were not told to look at any particular region of the luminance bar, but before performing the rewarded condition, they were informed that they could obtain a reward by looking at the low-salient region.

Participants started every trial by fixating a central cross and simultaneously pressing the space bar. After trial start, the central cross disappeared, and another fixation cross (first saccade target) appeared at the upper right corner of the screen (+14° to the right and +6° up relative to screen center). This first saccade target turned green once it had been fixated and the second saccade target (fixation cross) appeared horizontally centered and 6° up from the screen center. Once this second target had been fixated it turned green and the third saccade target (fixation cross) appeared horizontally centered and 6° down from the screen center. Likewise, the third saccade target turned green once it had been foveated. The luminance bar appeared 14° left or right from the last fixation cross with the high-salient region pointing up or down. It appeared with an onset delay of 0, 100, 200, 300 or 400 ms after gaze arrived at the last fixation cross. These onset delays were introduced to sample a broader range of reaction times. Thus, both conditions contained 40 trials of every onset delay, and trials with different onset delays were randomly interleaved. The vertical center of the luminance bar was aligned with the vertical center of the last fixation cross. The luminance bar and all three green fixation crosses disappeared 100 ms after the luminance bar had been foveated or after the overall trial duration of 5 s was exceeded.

In conditions with reward, feedback was provided at the end of each trial. Participants received nine score points for successfully looking at the rewarded region, otherwise, zero. This and their overall score (e.g., “+9|459”) was displayed at the final target location. Feedback was written in black, unless participants exceeded a time constraint of 5 s for the whole trial (red) or skipped a fixation cross (blue). Trials in which one or more of the three fixation crosses was skipped (3.98%) or in which the luminance bar was not foveated within the 5 s time constraint (additional 0.23%) were not considered for the final analysis.

##### Experiment 2 (continuous display)

Like Experiment 1, the continuous display experiment consisted of two conditions (unrewarded and rewarded), which were recorded in different blocks and contained 200 trials each (see Design and Procedure section of Experiment 1). Instructions were identical to instructions for Experiment 1. After starting a trial, the first saccade target (upper right fixation cross) appeared. It turned green once it was fixated, and, unlike Experiment 1, all subsequent fixation crosses and the luminance bar appeared (see Fig. 1b, second panel). Feedback was provided in the rewarded condition, identical to Experiment 1. We discarded 4.29% of trials because a fixation cross was skipped and additional 1.02% of trials because the time constraint was exceeded.

##### Experiment 3 (cued onset)

Experiment 3 consisted of two conditions: unrewarded and rewarded. Both conditions were split into two blocks of 200 trials each. For the first half of participants, it was recorded in different sessions (see Design and Procedure section of Experiment 1). For the other half of participants, it was recorded in one session together with Experiment 4. For these participants, the unrewarded condition was always recorded first, whereas the order of the remaining two blocks was balanced across participants. Instructions were identical to Experiments 1 and 2. Each block contained 20 trials for every combination of the five different onset delays and the two vertical bar positions (flipped vs. unflipped). Trials within one block were randomly interleaved.

Two cues validly indicated the location of the low-salient region (see Fig. 1c): (i) a dot presented for 250 ms above or below the initial fixation cross indicated whether the low-salient region was up or down relative to the last fixation cross, and (ii) the outline of the low-salient region, a black frame, was displayed once gaze was at the upper right fixation cross. The first cue was added to make sure performance was not limited by peripheral location discrimination performance. The second cue (outline) appeared together with the second and third saccade target and was displayed until the luminance bar appeared. The purpose of this second cue was to allow planning a saccade towards the low-salient region before the appearance of the luminance bar. The luminance bar appeared after foveating the last fixation cross with an onset delay of 0, 100, 200, 300, or 400 ms.

In order to avoid any implicit cue on the location of the high-salient region, the high-salient region could appear above or below the low-salient region. Therefore, the last fixation cross was vertically centered with the vertical bar like in all other experiments (unflipped), or it was vertically centered with the outer edge of the low-salient region (flipped). Feedback was provided in the rewarded condition. We discarded 4% of trials because a fixation cross was skipped and an additional 4.68% of trials because the 5 s time constraint was exceeded.

#### Data analysis

We measured eye movements of the right eye with a sampling rate of 1000 Hz. Saccade onsets and offsets were defined off-line using the EyeLink 1000 algorithm, which uses a combination of velocity (30°/s), acceleration (8,000°/s^2^) and motion (0.15°) threshold. The temporal difference between saccade onset and target onset was taken as saccade latency (Experiment 1) or Δ*t* (Experiment 3), respectively. Fixation durations (Experiment 2) were calculated as the temporal difference between the offset of the saccade foveating the final fixation cross and the onset of the subsequent saccade directing gaze to the luminance bar. An online criterium to detect saccade offsets and endpoints (10 frames on the target ±0.5° tolerance) was used to provide feedback. Conclusions did not change with the saccade offset criterion used. Endpoints were referenced to the vertical center of the last fixation cross, with positive values (<1.5°) indicating a response towards the low-salient region and negative values (>−1.5°) indicating a response towards the high-salient region. For trials of Experiment 3 in which the location of the salient region was vertically flipped (see Fig. 1c), values between 0° and 1.5° denote the low-salient region, whereas values between 1.5° and 3° denote the high-salient region.

To analyze endpoints over time, we used a cluster-based permutation approach, because dividing the data into bins did not allow to perform adequate statistics due to the variability across participants. Cluster-based permutation testing is successfully used in EEG where the data contain one time series per trial (Maris & Oostenveld, 2007). Recently, an equivalent method was introduced for behavioral data with one data point per trial (van Leeuwen, Smeets, & Belopolsky, 2019). Here, we used the method introduced by van Leeuwen et al. (2019): The data were first temporally smoothed for every individual; second, a weighted time series was constructed that takes the contribution of every individual into account, and third, a cluster-based permutation test was performed.

The data were smoothed using a Gaussian kernel of 16 ms width at a 1 ms resolution. For every time point of the smoothed data, two conditions were compared (or one condition against a baseline) using a *t* test. Clusters were defined as adjacent time points showing a significant difference between conditions with the cluster strength corresponding to the sum of the *t* values in the cluster. A cluster-based permutation approach was performed to determine cluster significance: For every permutation, the labels assigning trials to conditions were randomly shuffled. The permuted data was smoothed, and the weighted statistics yielded the strongest cluster indexed by the highest sum of *t* values. The cluster strength of the original, nonpermuted data (sum of *t* values) was then compared against the cluster-strength distribution of the strongest cluster for every permutation. Any cluster in the nonpermuted data with a cluster strength larger or equal to the 95th percentile of the permuted distribution was considered a significant cluster. The *p* value of a nonpermuted cluster is then given by 1 minus the percentile of the nonpermuted cluster in the permuted distribution (van Leeuwen et al., 2019). We used 10,000 permutations for every test. For every comparison, we report the *p* value, the cluster strength of the nonpermuted data (*t*) and the 95th percentile of the permuted distribution, which is the critical *t* value (*t*_*crit*_).

### Results

**Fig. 2:**
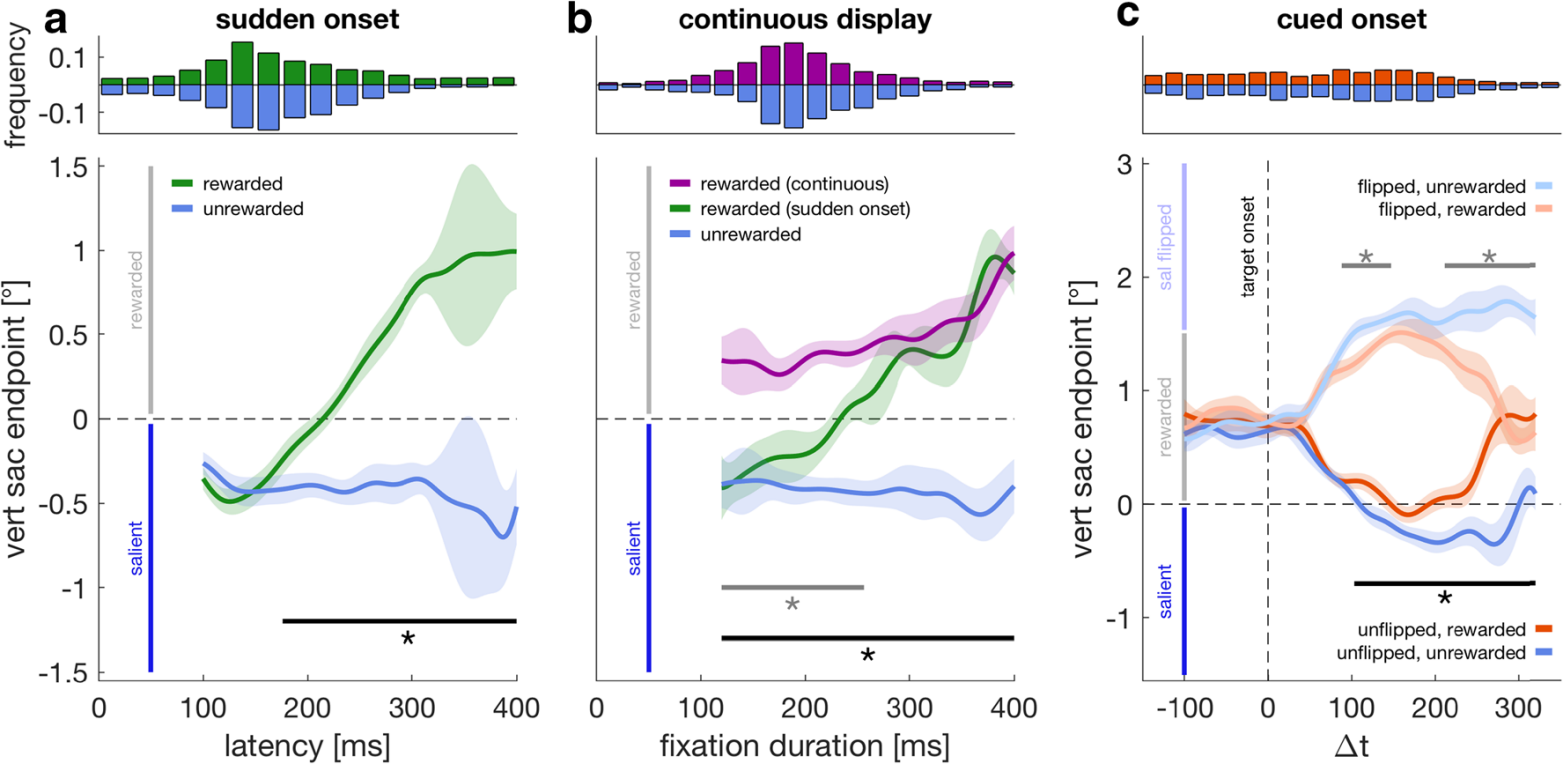
Vertical saccade endpoints (lower panels) for Experiments 1, 2, 3 with reaction time or fixation duration histograms (upper panels) for the respective conditions in the panel below. **a** Experiment 1 (sudden onset). Vertical saccade endpoints as a function of saccade latency when participants received a reward for looking at the low-salient region (green) or not (blue). The horizontal dashed line indicates the center of the luminance bar with positive values (<1.5°; vertical gray line) marking the rewarded region and negative values (> −1.5°; vertical blue line) marking the high-salient region. Data are smoothed weighted averages, with shaded regions being 95% confidence intervals (van Leeuwen et al., 2019). The solid horizontal black line and asterisk indicate a significant cluster in the respective time window. **b** Experiment 2 (continuous display). Vertical endpoints in the rewarded (purple) and unrewarded (blue) conditions as a function of the fixation duration on the final fixation cross. Green data are vertical endpoints from the rewarded sudden onset condition with 0 ms onset delay (**a**) expressed as a function of fixation duration. The comparison between rewarded continuous display and rewarded sudden onset shows that the difference between experiments is not an artifact of plotting endpoints as a function of latency versus fixation duration. The horizontal black line denotes a significant difference between the rewarded and unrewarded condition of Experiment 2, the gray line represents a significant difference between rewarded conditions of Experiment 1 and Experiment 2 and (**c**) Experiment 3 (cued onset). Vertical endpoints relative to target onset in the rewarded (orange) and unrewarded (blue) case. Faint colors denote trials in which the location of the salient region was flipped. Before target onset (Δ*t* < 0), the outline of the low-salient region was displayed as a cue. Confidence intervals of all depicted end-point time courses are a weighted statistic that take into account the individual weight given to each time point in the two conditions as well as the difference between two conditions across time for every participant (van Leeuwen et al., 2019). Consequently, when comparing two conditions, both confidence intervals are identical. All error bars result from comparing a rewarded with the corresponding unrewarded condition, unless the sudden onset data in **b**, which results from a comparison with the rewarded continuous data. (Color figure online)

In Experiment 1, we aimed to replicate the dynamic transition from salience to reward in a saccade sequence instead of a single reactive saccade. In this experiment (see Fig. 1a), the next target in a sequence only appeared once the previous target had been fixated. The lower panel in Fig. 2a (see individual data in Supplemental Fig. S1) shows vertical saccade endpoints as a function of saccade latency (i.e., saccade onset relative to target onset) when the low-salient region was rewarded (green) or not (blue). Whereas without reward endpoints were always biased towards salience, endpoints in the reward condition critically depended on saccade latency: Early saccades were biased by salience, later saccades were biased by reward. The two time courses differed significantly for latencies of 176 ms or longer (*p* < .0001, *t* = 1554, *t*_*crit*_ = 147.2).

In Experiment 2, we measured the same sequence with all targets being continuously displayed throughout the trial (see Fig. 1b). Figure 2b shows vertical endpoints as a function of the fixation duration on the final fixation cross (i.e., the fixation before the saccade to the target area was made; see individual data in Supplemental Fig. S2). Again, without reward, endpoints were biased towards salience throughout the whole time course (blue). With reward, endpoints were biased towards the rewarded region for all fixation durations (120–400 ms, purple line) and were different from endpoints without reward (*p* < .0001, *t* = 2419, *t*_*crit*_ = 148.7). Thus, there was no transition from salience to reward. To ensure that this was due to the continuous presence of the target throughout the trial and not an artifact of plotting endpoints as a function of fixation duration instead of latency, we also plotted the data from the rewarded sudden onset condition, with 0 ms onset delay as a function of fixation duration (see green data in Fig. 2b). With reward, vertical endpoints in the continuous and sudden onset condition differed significantly for fixation durations below 257 ms (*p* < .0001, *t* = 624.09, *t*_*crit*_ = 183). Thus, we observed a transition in vertical endpoints from salience to reward when the target suddenly appeared before the saccade was initiated, but not when the target was continuously displayed throughout the sequence.

Experiments 1 and 2 differ with regard to two aspects: when the target is continuously displayed (Experiment 2), participants can know at the beginning of the sequence where they want their eyes to land and which location to inhibit. Neither is possible in Experiment 1. In Experiment 3 we measured eye movements in the same saccade sequence when participants knew from the beginning where to look, but not which location to inhibit (see Fig. 1c). Therefore, the outline of the low-salient (rewarded) region was visible before the luminance bar appeared. However, the salient region could appear above or below.

Figure 2C (see individual data in Supplemental Fig. S3) shows vertical saccade endpoints in Experiment 3 as a function of the temporal difference between saccade onset and target onset (Δ*t*). Please note that due to the different onset delays, it was possible to initiate a saccade to the luminance bar before it appears (i.e., to the outline of the low-salient region), resulting in a negative Δ*t*. In all conditions, saccades accurately landed in the highlighted outline when they were initiated before the target appeared. In contrast, endpoints of saccades initiated after target onset strongly depended on the actual temporal difference between saccade onset and target onset. Shortly after target onset, endpoints were drawn towards salience: Endpoints to flipped (faint colors) and unflipped targets (saturated colors) differed both with reward (orange; *p* < .0001, 50–260 ms, *t* = 2129, *t*_*crit*_ = 139.9) and without (blue; *p* < .0001, ≥42 ms, *t* = 4549, *t*_*crit*_ = 148.6). In the unflipped case, endpoints with and without reward differed significantly for Δ*t* values above 102 ms (*p* = .0001, *t* = 767.8, *t*_*crit*_ = 153.3). In the flipped case, we observed two significant time clusters, an early (88–147 ms, *p* = .0211, *t* = 177.9, *t*_*crit*_ = 146.8) and a late one (211–320, *p* < 0.0001, *t* = 606.5, *t*_*crit*_ = 146.8). Both in the flipped and unflipped case, endpoints were drawn back to the center of the rewarded region. Thus, endpoints were biased towards salience, although participants knew in advance where to look and had sufficient time to integrate that information into their saccade plan.

### Discussion

Experiments 1, 2, 3 were designed to dissociate whether the transition from bottom-up to top-down oculomotor control (see Fig. 2a) is determined by the time it takes to integrate information about behavioral goals into the saccade plan (planning hypothesis) or by the time it takes to inhibit a response to a suddenly appearing salient stimulus (inhibition hypothesis). Our results support the latter. We showed that early responses are not necessarily governed by salience, but that this depends on whether the target can be previewed or not (see Fig. 2b). More importantly, when participants had sufficient time to plan a saccade to the low-salient rewarded region, an unpredictable onset still temporarily biased endpoints towards salience (see Fig. 2c).

In Experiment 1 every target appeared once the previous target had been fixated, and the location of the luminance bar could not be anticipated before it appeared. Therefore, it is possible that sudden onsets only attracted saccades, because they were relevant for the task. However, this was not the case in Experiment 3. In this experiment, all subsequent fixation crosses and the outline of the low-salient region did not appear one after the other, but once the first saccade target was fixated. Moreover, in Experiment 3 the onset of the luminance bar was not relevant to perform the task. This supports the notion that the onset of a salient stimulus not only affected saccades when it was task-relevant, but because of its physical salience.

In our experiments, we did not manipulate the relative salience of the two regions. Changing, for example, the salience of the high-salient region would have changed how much early responses in the sudden onset experiment were drawn towards the salient region (Schütz et al., 2012). Therefore, changing the relative salience of the luminance bar would have most likely changed the magnitude of the onset effect—that is, how much the two orange curves in Fig. 2c were maximally drawn apart before inhibition became apparent and endpoints were redirected towards the center of the rewarded region.

An alternative explanation to actively inhibiting a response to the suddenly appearing salient region would be that salience is only briefly presented in the brain and automatically decays over time. However, we consider it more likely that a response to the salient region has to be actively inhibited for two reasons. The first reason is the recent converging evidence that emphasizes the role of active inhibition to prevent attentional capture by salient stimuli (for reviews, see Gaspelin & Luck, 2018, 2019; van Moorselaar & Slagter, 2020)*.* The second reason is the observed pattern in our Experiment 3 (cued onset): In both conditions, saccade responses initiated before target onset were reliably aimed at the center of the low-salient (rewarded) region. After target onset, endpoints in the rewarded condition were first drawn towards salience, but then regressed back to the center of the rewarded region (orange lines in Fig. 2c). This pattern would be consistent with both automatic decline and active inhibition. However, if this was caused by an automatic decline, we would have expected to find the same pattern without a behavioral goal, thus in the unrewarded condition (blue lines in Fig. 2c). Yet this was not the case: Endpoints after target onset were initially biased towards salience, but then remained at the salient region and did not return to the center of the low-salient region although a saccade to that location had already been planned. We take this as evidence for active inhibition. In Experiments 4, 5, 6, we study the requirements and limitations of this inhibition.

## Experiment 4: Fully predictable onsets bias endpoints towards salience despite correction

What determines whether a response to a suddenly appearing salient stimulus can be successfully inhibited or not? In Experiment 4 (predictable onset experiment), we tested whether a bias by a suddenly appearing salient stimulus can be inhibited when the onset is fully predictable. To this end, we measured a slightly modified version of Experiment 3 in which the luminance bar was always vertically aligned with the center of the last fixation cross (i.e., unflipped trials only). As a consequence, the two cues were informative not only about the location of the low-salient rewarded region but also about the location of the high-salient region. We provided this information explicitly to participants before the experiment. If successful inhibition can be achieved by making the stimulus onset fully predictable, we expected endpoints to be centered on the rewarded region independent of the temporal difference between saccade and target onset. On the other hand, if inhibition cannot be achieved by onset anticipation, we expected endpoints after target onset to be biased towards salience by the same extent as in Experiment 3.

### Methods

We recorded data of 16 participants (mean age = 24 years, age range: 18–30 years, 14 females) for Experiment 4. All participants also took part in Experiment 3. Twelve participants performed Experiment 4 in one session together with Experiment 3 (see Design and Procedure section of Experiment 3), the remaining four participants performed Experiment 4 in a separate session.

Experiment 4 consisted of one condition (rewarded) that was recorded in one block containing 200 trials. Trials were identical with rewarded unflipped trials (see Fig. 1c) from Experiment 3. Thus, in Experiment 4, the center of the luminance bar was always vertically aligned with the last fixation cross such that the outline of the rewarded region was also informative about the salient region’s location. We provided this information explicitly to the participants. Like in Experiment 3, the luminance bar could appear with an onset delay of 0, 100, 200, 300, or 400 ms after the last fixation cross was foveated. Trials with different onset delays were randomly interleaved in one block. We discarded trials in which a fixation cross was skipped (3.97%) or in which the overall trial duration of 5 s was exceeded (additional 0.25%) for the final analysis.

Data analysis for endpoints over time was equivalent to Experiments 1, 2, 3. We compared observed and optimal aimpoints (see Results) using a paired-samples *t* test, complemented with the Bayesian equivalent (Wagenmakers et al., 2017). The Bayesian *t* test yields a Bayes factor, BF_10_. BF_10_ values <1 favor the null hypothesis (no difference between samples), and BF_10_ values >1 favor the alternative hypothesis (difference between conditions). The more values deviate from 1, the stronger the evidence, with BF_10_ values in between 0.33 and 3 being considered inconclusive evidence (Jeffreys, 1961).

### Results and discussion

**Fig. 3.**
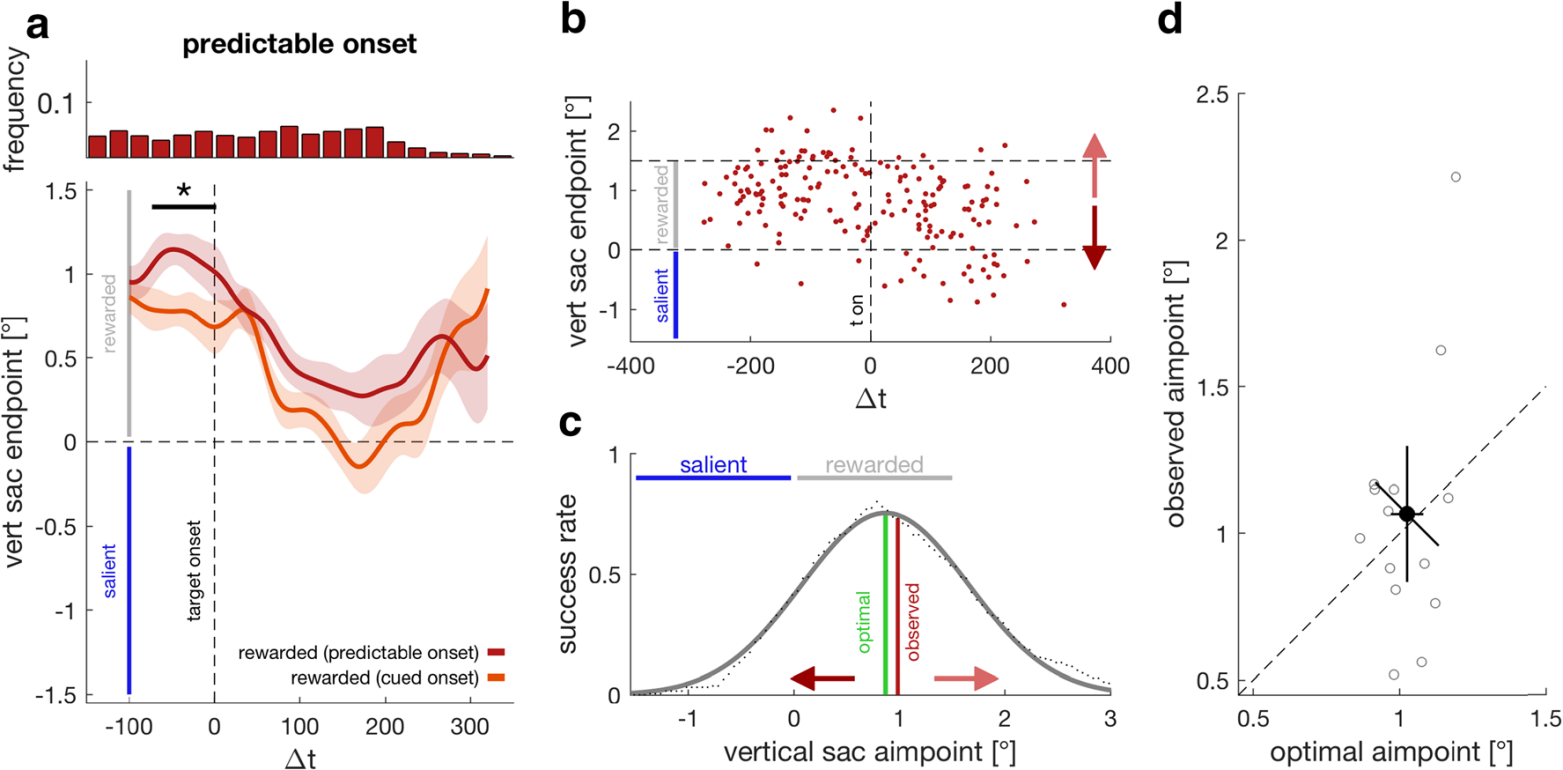
Experiment 4 (predictable onset). **a** Vertical saccade endpoints relative to target onset (red). The orange time course represents data from unflipped trials of Experiment 3 from the same set of participants. The upper panel shows a reaction time histogram for the predictable onset data below. **b** Data of one participant. Red dots denote endpoints of individual trials. The aimpoint was defined as the average endpoint before target onset (Δ*t* < 0). Trials with endpoints in between the two horizontal dashed lines were rewarded. The fraction of rewarded trials yields the success rate. We computed the success rate for different theoretical aimpoints by shifting all data points up and down (red vertical arrows). **c** Success rate over different aimpoints for the same observer as in **b**. Black dots are the success rate for a given aimpoint and thus for a given vertical shift of all endpoints. The solid gray line is a Gaussian fit of the success rates over different aimpoints. The vertical red line shows the actually observed success rate and aimpoint, whereas the green vertical bar denotes the optimal aimpoint maximizing the success rate and thus the reward outcome. The optimal aimpoint is defined by the mode/mean of the Gaussian. **d** Observed against optimal aimpoints. Open circles denote data from individual participants, and the filled circle is the group average with 95% confidence intervals of between-participant variability. (Color figure online)

Figure 3a shows endpoints from Experiment 4 (red) compared with unflipped trials from Experiment 3 (orange) for the same set of participants (see individual data in Supplemental Fig. S4). We found one significant cluster from −73 to +2 ms (*p* = .0087, *t* = 233.5, *t*_*crit*_ = 157.6), highlighting that participants aimed to saccade to a location further away from the salient region. The aimpoint (mean vertical endpoint for Δ*t* < 0) was 1.065° (*SD* = 0.42°). Endpoints after target onset were different from this initial aimpoint for Δ*t* values >33 ms (*p* < .0001, *t* = 1305, *t*_*crit*_ = 149.7). Thus, endpoints after target onset were still drawn in the direction of salience. To assess whether this aimpoint adjustment is functional, we compared actual aimpoints with the aimpoint that would have maximized the rate of successfully looking at the rewarded region. Therefore, we shifted the whole end-point distribution of each participant up or down (see Fig. 3b) and computed the fraction of trials within the rewarded region (success rate) for every possible aimpoint. Success rates over the different aimpoints were fitted with a Gaussian, the mean of which was taken as optimal (see Fig. 3c). Observed aimpoints (see Fig. 3d) were not different from optimal aimpoints (1.025°), *t*(14) = 0.405, *p* = .691, *BF*_10_ = 0.282.

In Experiment 4, we asked whether it is possible to prevent being biased towards salience when one knows both where to look and which location to inhibit. To this end, we made use of the same two cues used in Experiment 3. But unlike Experiment 3, both cues were also informative about the salient region’s location, and we provided this information explicitly to participants. The results showed that when participants know where to look and which location to inhibit, they initially correct for the sudden onset of a salient stimulus, but they cannot prevent being affected by it. This suggests that full knowledge about the onset of a suddenly appearing salient stimulus is not sufficient for successful inhibition.

## Experiment 5: Preview quality modulates the inhibition of salience

**Fig. 4:**
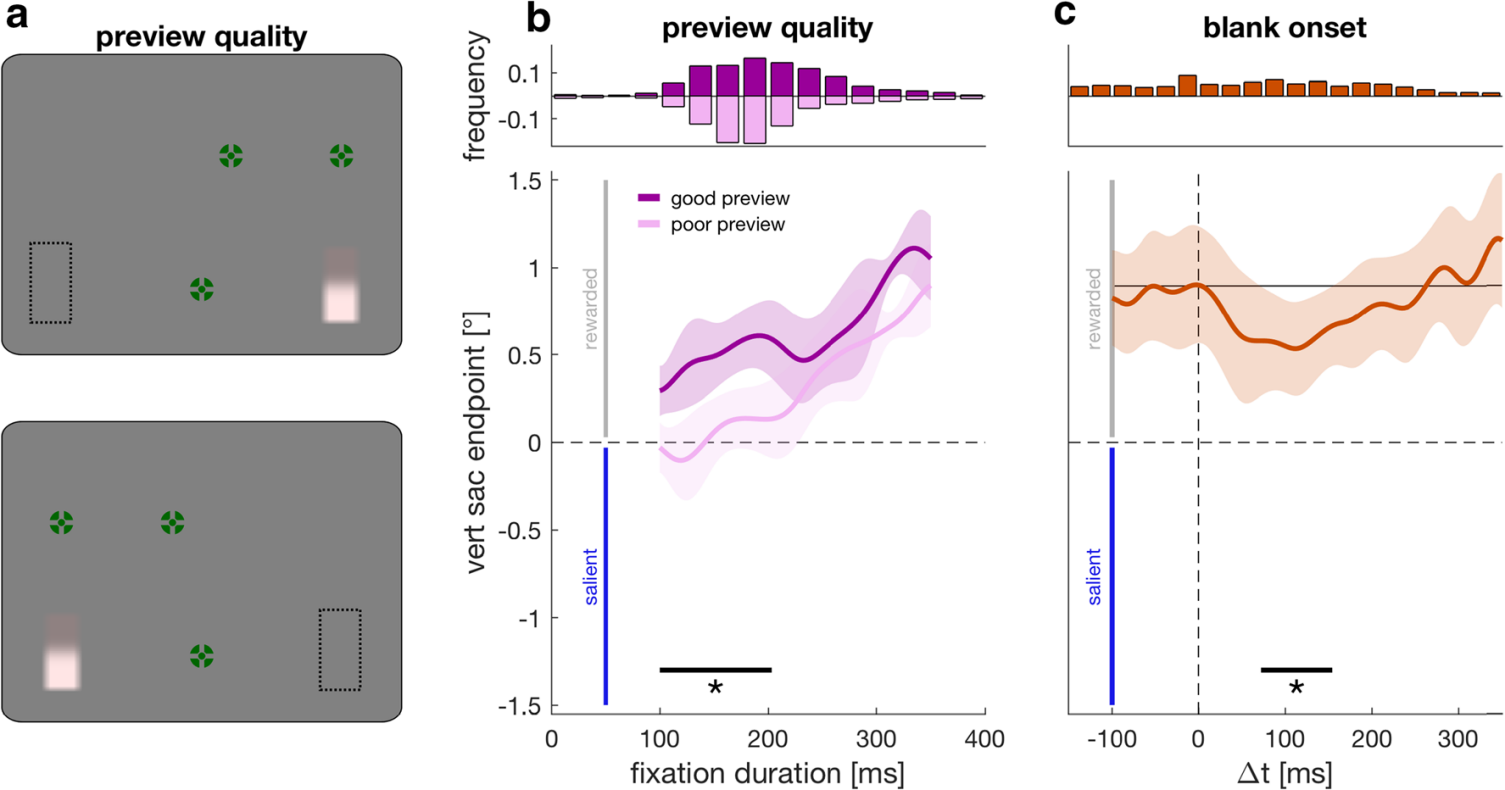
Experiment 5 (preview quality; **a, b**) and Experiment 6 (blank onset; **c**). **a** Spatial layout of saccade targets in Experiment 5. Upper panel: Once the first saccade target (upper right fixation cross) was fixated, all remaining saccade targets appeared. Like in all other experiments, fixation crosses turned from black to green once fixated. The location of the first two fixation crosses was changed so that they were closer to the final target if the luminance bar appeared on the right (good preview location) compared with when it appeared on the left (poor preview location). The dashed outline corresponds to the target location in bad preview trials and was not displayed during the experiment. Lower panel: For half of the participants the location of the fixation crosses was horizontally mirrored to prevent a confound between preview quality (good vs. poor) and saccade direction of the final saccade (left vs. right). **b** Vertical endpoints as a function of fixation duration for the good (saturated) and poor (faint) preview conditions. **c** Vertical endpoints relative to target reappearance (Δ*t* = 0) after the target was blanked. The solid horizontal line marks the average endpoint for saccades with Δ*t* < 0. Shaded regions represent the 95% confidence interval (van Leeuwen et al., 2019). (Color figure online)

As an alternative to anticipatory inhibition, it might be that successful inhibition requires visual processing of the target in which case performance should depend on how well a target can be previewed in the periphery. If this were the case, we would expect that successful inhibition is modulated by how well the target can be seen before a response is made. In Experiment 5 (preview quality experiment) we thus investigated whether inhibition is modulated by visual preview quality. To test this, we manipulated the eccentricity with which the target can be peripherally inspected during the saccade sequence. This was achieved by changing the location of the upper two fixation crosses (saccade target 1 and 2) so that luminance bars appearing in one hemifield were closer to these two fixation crosses than were bars in the other hemifield (see Fig. 4a). If inhibition is modulated by preview quality, we would expect that responses towards the good preview location are more clearly directed into the rewarded region. This should be particularly pronounced when the fixation duration on the last fixation cross (equal distance to both locations) is short.

### Methods

We recorded data from a new set of eight participants (mean age = 25 years, age range: 20–34 years, one male). Every participant completed two blocks of 200 trials each. Each block contained 100 trials with good and 100 trials with poor preview, which were randomly interleaved within a block. In both blocks, participants received a reward for saccades into the low-salient region.

Trial procedure was identical to the rewarded condition of Experiment 2, except for two changes: (i) the overall trial duration was restricted to 2.3 s (instead of 5 s) to have shorter fixation durations, and (ii) the location of the first two saccade targets (i.e., the upper two fixation crosses) was changed: The vertical position of these first two saccade targets was 3.5° above screen center (instead of 6°). Moreover, the horizontal position of the second saccade target was horizontally shifted in the direction of the first saccade target by 2.5°. As a consequence, the difference in overall eccentricity between these fixation crosses and the luminance bar became more pronounced for luminance bars appearing on the right compared with bars appearing on the left (see Fig. 4a). Specifically, the overall distance of the upper right fixation cross to the luminance bar was 9.5° (instead of 12°) if the luminance bar appeared on the right and 29.6° (instead of 30.5°) if the luminance bar appeared on the left. The distance of the second saccade target to the luminance bar was 14.9° for a luminance bar on the right and 19.3° for a luminance bar on the left (compared with 18.4° to both locations in all other experiments).

Preview quality would be confounded with saccade direction when the good preview target always appeared on the right and the poor preview target always appeared on the left. Therefore, the sequence was horizontally mirrored for half of the eight participants (Fig. 4a, lower panel). Thus, for these participants the first saccade target always appeared on the upper left and not on the upper right, and the second saccade target was shifted 2.5° to the left and not to the right. We discarded trials in which at least one fixation cross was skipped (8.81%) or in which the luminance bar was not fixated within the time constraint of 2.3 s (additional 0.75%).

### Results and discussion

Figure 4b shows vertical endpoints for the good and poor preview conditions as a function of the fixation duration on the last fixation cross (see individual data in Supplemental Fig. S5). For fixation durations below 203 ms, saccades to the side with poor preview quality were further away from the rewarded region than saccades to the side with good preview quality (*p* = .0021, *t* = 291, *t*_*crit*_ = 144.4).

These results show that saccades to targets appearing in a hemifield with good preview were more reliably aimed towards the rewarded region. This became evident when the fixation on the final fixation cross was short. The results suggest that successfully inhibiting the salient region depends on preview quality and thus that it requires visual processing of the target. In a next step, we wanted to know what happens if inhibition has been successfully established and the target temporarily disappears. Can it be maintained? Or does it decay once the target is removed from the screen?

## Experiment 6: Target reappearances bias endpoints towards salience

Experiment 6 (blank onset experiment) aimed to test whether inhibition can be maintained if the target is blanked and thus reappears shortly before the final response is made. To this end, the luminance bar could be sufficiently previewed in its final location and orientation before the saccade sequence was carried out. However, in half of the trials, the target was blanked during the saccade sequence and only reappeared when the gaze arrived at the final fixation cross or shortly after.

### Methods

We recruited another new set of 16 participants. Data from two of the 16 participants had to be discarded because they stated after the experiment that they were not able to identify the feedback or peripherally identify the orientation of the luminance bar. The remaining 14 participants (12 females) had a mean age of 24 years (age range: 20–30 years).

Experiment 6 consisted of 400 trials recorded in two blocks of 200 trials each. Each block contained 100 trials with blank and 100 trials without blank. These two trial types were randomly interleaved. Trials without blank were interleaved to make the blank less predictable and ensure that participants pursued a steady pace in the saccade sequence. In all trials, participants received a reward for saccades into the low-salient region.

The trial procedure of Experiment 6 is graphically depicted in Supplemental Fig. S6. The vertical bar appeared 1.2 s before the initial central fixation cross was removed and the fixation cross in the upper right appeared. Participants were instructed to keep fixating the central fixation cross during that time (eccentricity of 15.2°), and the vertical bar was temporarily removed from the screen in case participants shifted their gaze away from the central fixation cross. As soon as the upper right fixation cross had appeared and was foveated, the other two fixation crosses appeared and remained on the screen throughout the trial (only changing their color once they had been fixated)

In half of the trials, the vertical bar was blanked 100 ms after the upper central fixation cross was fixated, and it reappeared with one of the onset delays also used in Experiments 1, 3, and 4 (0, 100, 200, 300, and 400 ms) after the next and last fixation cross was fixated. In the other half of the trials, the bar was not blanked. Trials in Experiment 6 had a time constraint of 3.5 seconds upon appearance of the upper right fixation cross. We discarded 3.54% of trials because a fixation cross was skipped and additional 0.39% of trials because the time constraint was exceeded.

### Results and discussion

Figure 4c shows vertical endpoints on the luminance bar relative to target reappearance (see individual data in Supplemental Fig. S7). The average endpoint for saccades initiated before target reappearance was 0.9° (*SD* = 0.52°). Saccades initiated between 72 and 154 ms after target reappearance were significantly drawn towards salience (*p* = .012, *t* = 217.08, *t*_*crit*_ = 157.7).

Thus, even if the target can be previewed for a sufficient duration, but is blanked and thus reappears shortly before the final saccade to the luminance bar is made, the reappearance of salient stimuli biases endpoints, suggesting that inhibition cannot be fully maintained.

## General discussion

Selecting a visual object as a target for attention or an eye movement is determined by bottom-up and top-down factors. Early responses are biased towards salience, and only late responses can be governed by top-down control. However, this is only true when a stimulus cannot be previewed and appears suddenly in the periphery (see Fig. 2b). In this study, we show that deliberate planning is insufficient for an early transition to top-down control and that the inhibition of suddenly appearing salient stimuli is an additionally required separate process. Even with sufficient time dedicated to saccade planning, the sudden onset of the target biased saccade endpoints towards salience (Fig. 2c). The time it takes to fully suppress the salient region is comparable whether there was sufficient time to preplan the saccade (Fig. 2c) or not (Fig. 2a), suggesting that although target selection is required, the transition from bottom-up to top-down control will ultimately be determined by the process of inhibiting the suddenly appearing salient region. Even prior knowledge about both, where to look and which location to inhibit, is not sufficient. It causes an anticipatory compensation but cannot prevent being drawn towards salience (see Fig. 3a). Successful inhibition could only be achieved by previewing the target (Fig. 4b). However, simply blanking the target briefly biased saccade endpoints in the direction of salience again, even with a sufficiently long preview duration (see Fig. 4c), suggesting that inhibition cannot be fully maintained when the target is blanked. Yet this onset bias appears comparatively small compared with other conditions in which the target cannot be previewed, but where the location of the target and salient region are known (see Figs. 2c and 3a).

Our participants had to make a sequence of saccades with the last target being a vertical luminance bar consisting of a high-salient and a low-salient region. We used reward to manipulate behavioral goals. Without reward (i.e., without a behavioral goal), endpoints were aimed at the salient region. This was also true when the outline of the low-salient region was shown, and the saccade was already planned (see Fig. 2c). This indicates that without a behavioral goal, it is the default to select salience (Schütz et al., 2012; Wolf, Wagner, & Schütz, 2019), and this default bias does not decay over time but needs to be actively suppressed (Gaspar & McDonald, 2014; Gaspelin, Leonard, & Luck, 2015) when salience and behavioral goals compete. In contrast to that, Donk and van Zoest (2008) found that correct saccade selection decreased with increasing latency when participants had to select the most salient target. However, in their study, two salient targets competed (Donk & van Zoest, 2008), and the performance decay might reflect the influence of the second salient target, which is exerted at a later time point. This could be reflected in the timing of the posterior contralateral negativity (PCN), an event-related potentials related to attention and visual capture, which is negatively correlated with target salience (Töllner, Zehetleitner, Gramann, & Müller, 2011).

In our study, the distractor (i.e., the salient region) was part of the same object and therefore always appeared in close spatial proximity to the target. Distractor appearing close to the target can decrease saccade latencies (Briand, Larrison, & Sereno, 2000; Khan, Heinen, & McPeek, 2010; Khan, Munoz, Takahashi, Blohm, & McPeek, 2016) and might therefore additionally increase the capture by a sudden-onset stimulus. On the contrary, a distractor appearing further away from a prespecified saccade target can increase saccade latency leading to a remote distractor effect (Ludwig, Gilchrist, & McSorley, 2005; Walker et al., 1997). It is possible that our sudden onset manipulation also affected saccade timing. However, our data cannot reveal if onsets increased or decreased saccade latencies, especially since we lack an informative control condition. Sudden distractor onsets can also give rise to saccadic inhibition that is characterized by a dip in the latency distribution around 70–100 ms after distractor onset (Bompas, Campbell, & Sumner, 2020; Bompas & Sumner, 2015; Buonocore & McIntosh, 2008; Edelman & Xu, 2009; Reingold & Stampe, 1999, 2002). Saccadic inhibition is thought to arise from competing activation in saccade planning areas like the superior collicus (SC; Dorris, Olivier, & Munoz, 2007; Meeter, Van der Stigchel, & Theeuwes, 2010; White et al., 2013). We did not observe any unequivocal evidence for saccadic inhibition in experiments where we measured endpoints time locked to a suddenly appearing stimulus. The absence of a clear dip around 100 ms after a sudden onset can have several possible reasons: For example, in our experiments, distractor onset and the potential start of saccade planning have been separated by up to several seconds. Moreover, saccadic inhibition is typically studied with spatially distinct distractors or changes to the full visual display, whereas in our paradigm, target and distractor were parts of the same object. Relatedly, the activation of top-down signals in SC is suppressed by sudden distractor onsets, but rebounds if the distractor is spatially close to the target (White et al., 2013). This suggests that our findings might be restricted to sudden onsets close to a prespecified saccade goal, but also render the SC as a possible neural origin.

Attention and gaze can be captured not only by suddenly appearing stimuli but also by other salient targets—for example, a color or a form singleton (Theeuwes, 1992) or by targets sharing a feature with a designated target, irrespective of their physical salience (Folk, Remington, & Johnston, 1992; Leber & Egeth, 2006). Recently, it was proposed that these seemingly opposing viewpoints of either bottom-up or top-down capture might be related by a suppressive mechanism (Gaspelin & Luck, 2018, 2019): salient stimuli have the capability of capturing attention, but can be voluntarily inhibited. This suppressive mechanism is thought to be reflected in the distractor positivity (P_D_) component of the event-related potential, which can be observed when distractors fail to capture attention (Gaspar & McDonald, 2014; Sawaki & Luck, 2010). If a salient item is successfully inhibited as indexed by a P_D_ component, then these items cause no preceding attention shift, which is reflected is the absence of a N2pc component (Gaspelin et al., 2015; Sawaki & Luck, 2010). This ERP pattern was taken as evidence that distractors can be proactively inhibited (for reviews, see Gaspelin & Luck, 2018, 2019; van Moorselaar & Slagter, 2020). In contrast to that, in our paradigm, participants were not able to proactively inhibit being biased towards the suddenly appearing salient region, even when both the location of the intended saccade target and the salient location were known in advance (see Fig. 3a). Yet the fact that endpoints before target onset shifted into the rewarded region and away from the salient region shows that participants made use of that information. Our results suggest that sudden onset stimuli cannot be proactively suppressed, but that suppression requires a preview of the target as suggested by the process of visual marking (Watson & Humphreys, 1997; Watson, Humphreys, & Olivers, 2003). Visual marking is typically studied using visual search tasks by delaying the onset of a group of items. It is supposed to aid selection in time by collectively rejecting old items using a location-based inhibitory template and thereby increasing search efficiency (Watson et al., 2003). Donk and Theeuwes (2001) argued that visual marking can be explained by the abrupt onset of luminance-defined targets, because no such preview benefit can be found under isoluminant conditions. Our results are consistent with both viewpoints: The abrupt onset of the luminance bar biased endpoints in the direction of the salient region (i.e., the region with the higher luminance contrast), even when the target location was previewed and this old item was thus not prone to rejection (see Fig. 2c). However, previewing the luminance bar and the to-be-rejected salient region appeared to reduce the bias caused by the onset of the luminance bar (Fig. 4b compared with Fig. 2c).

Preparatory suppression might have been possible if locations were not balanced and participants were given the chance to learn statistical regularities of target and distractor (Ferrante et al., 2018; Wang & Theeuwes, 2018a, 2018b). The suppression of a distractor might be achieved by different mechanisms and thus reflected in different markers depending on task, context, and stimulus material (for reviews, see Chelazzi, Marini, Pascucci, & Turatto, 2019; Noonan, Crittenden, Jensen, & Stokes, 2018). For example, when one target is preferred over the other based on its location/hemifield, a pretarget lateralization in alpha power is observed (Heuer, Wolf, Schütz, & Schubö, 2017), whereas no alpha power lateralization is observed when targets are preferably selected because of a feature (Heuer, Wolf, Schütz, & Schubö, 2019), although both tasks yield a similar pattern of behavioral results. A recent study linking ERP markers and single-unit activity in FEF (Cosman, Lowe, Woodman, & Schall, 2018) revealed that FEF activity precedes ERP signals and that FEF, like LIP (Ipata, Gee, Gottlieb, Bisley, & Goldberg, 2006), contributes to target selection and distractor suppression. Strikingly, target selection and distractor suppression were achieved by overlapping neural populations. Given the retinotopic nature of the FEF, this might explain why sudden onset stimuli appearing in close spatial proximity to the saccade target are successful in affecting gaze and cannot be proactively suppressed.

To conclude, when salience and behavioral goals compete for oculomotor selection, early saccades are biased towards salience, unless the target can be adequately previewed. Deliberate planning is thus not sufficient for successful top-down control of eye movements. Additional inhibition of suddenly appearing salient stimuli is necessary, highlighting the importance of inhibition for top-down control of human eye movements.

## Electronic supplementary material

ESM 1 (PDF 2100 kb)
