# Supplementary material for: Top-down control of saccades requires inhibition of suddenly appearing stimuli
Source: Atten Percept Psychophys. 2020 Aug 16;82(8):3863–77. doi: 10.3758/s13414-020-02101-3 (PMC7593282; doi:10.3758/s13414-020-02101-3)
Supplement: Supplementary file 1 — (PDF 2100 kb) [file 13414_2020_2101_MOESM1_ESM.pdf]

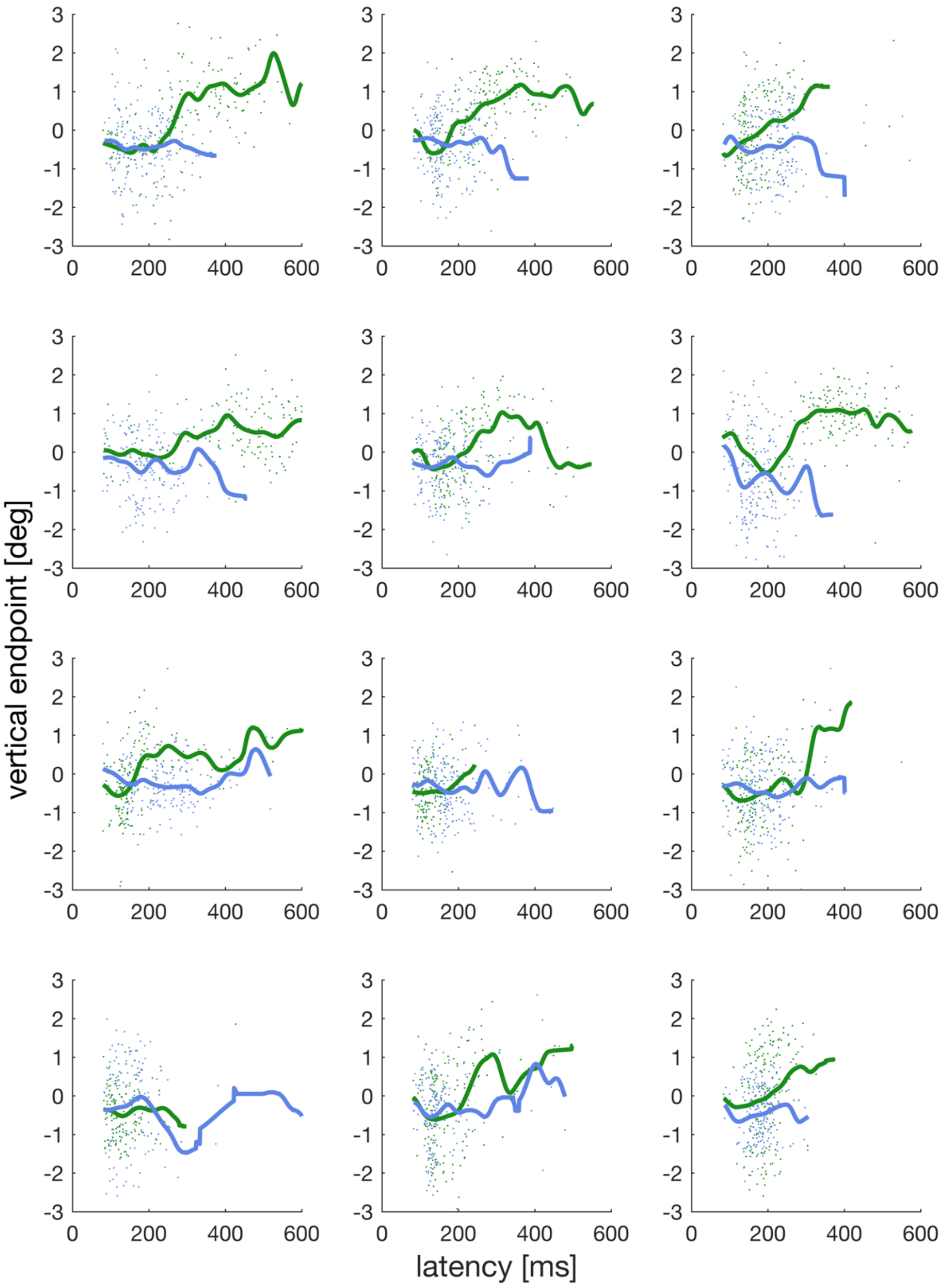

Suppl. Fig. S1. **Individual data Experiment 1 (sudden onset).** Vertical endpoints as a function of saccade latency for rewarded (green) and unrewarded trials (blue). Every panel denotes one participant, every data point denotes an individual trial. Lines are weighted averages, smoothed by a Gaussian window with a standard deviation of 16 ms.

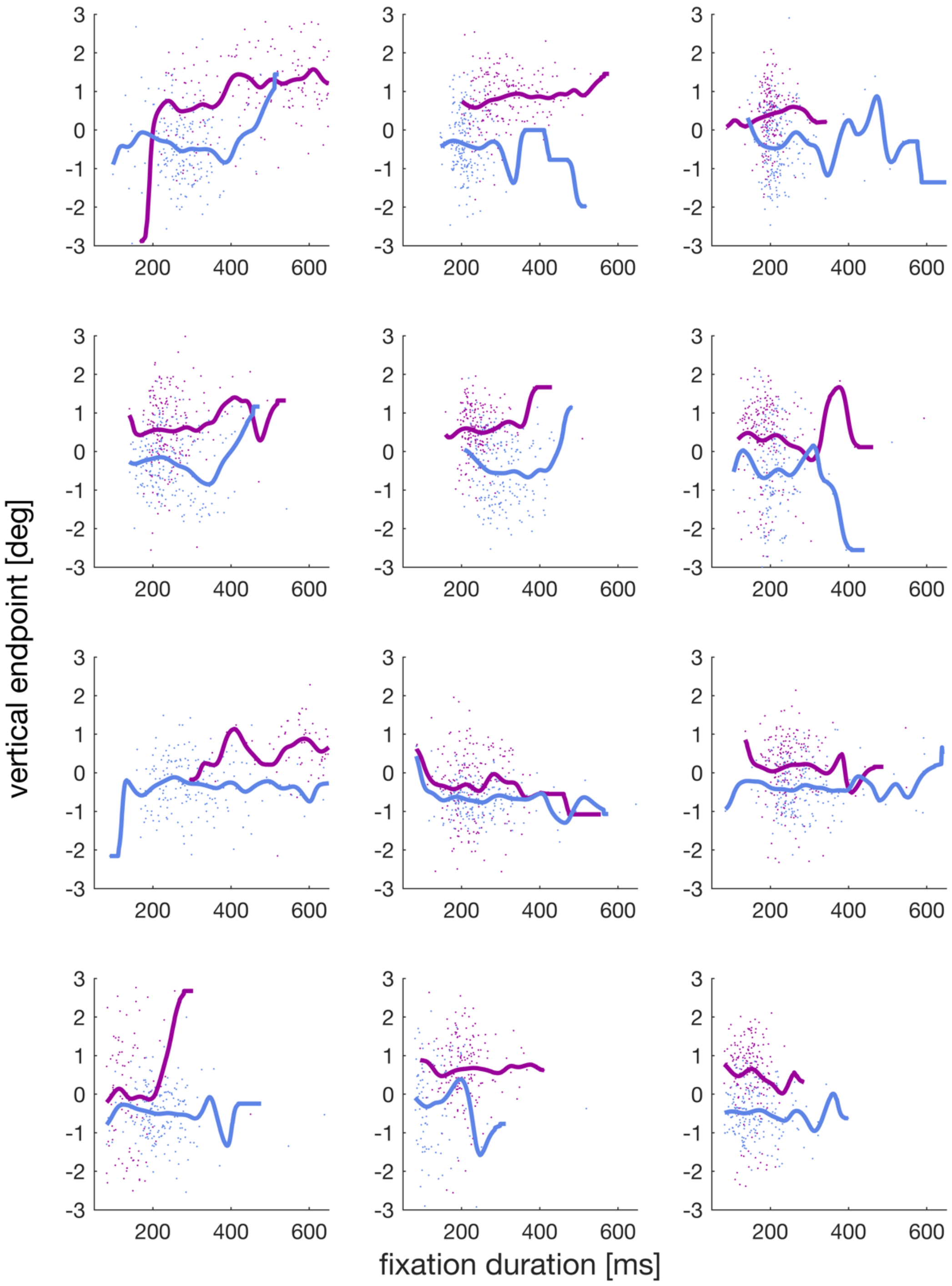

Suppl. Fig. S2. **Individual data Experiment 2 (continuous)**. Vertical endpoints as a function of fixation duration for rewarded (purple) and unrewarded trials (blue). Every panel denotes one participant, every data point denotes an individual trial. Lines are weighted averages, smoothed by a Gaussian window with a standard deviation of 16 ms.

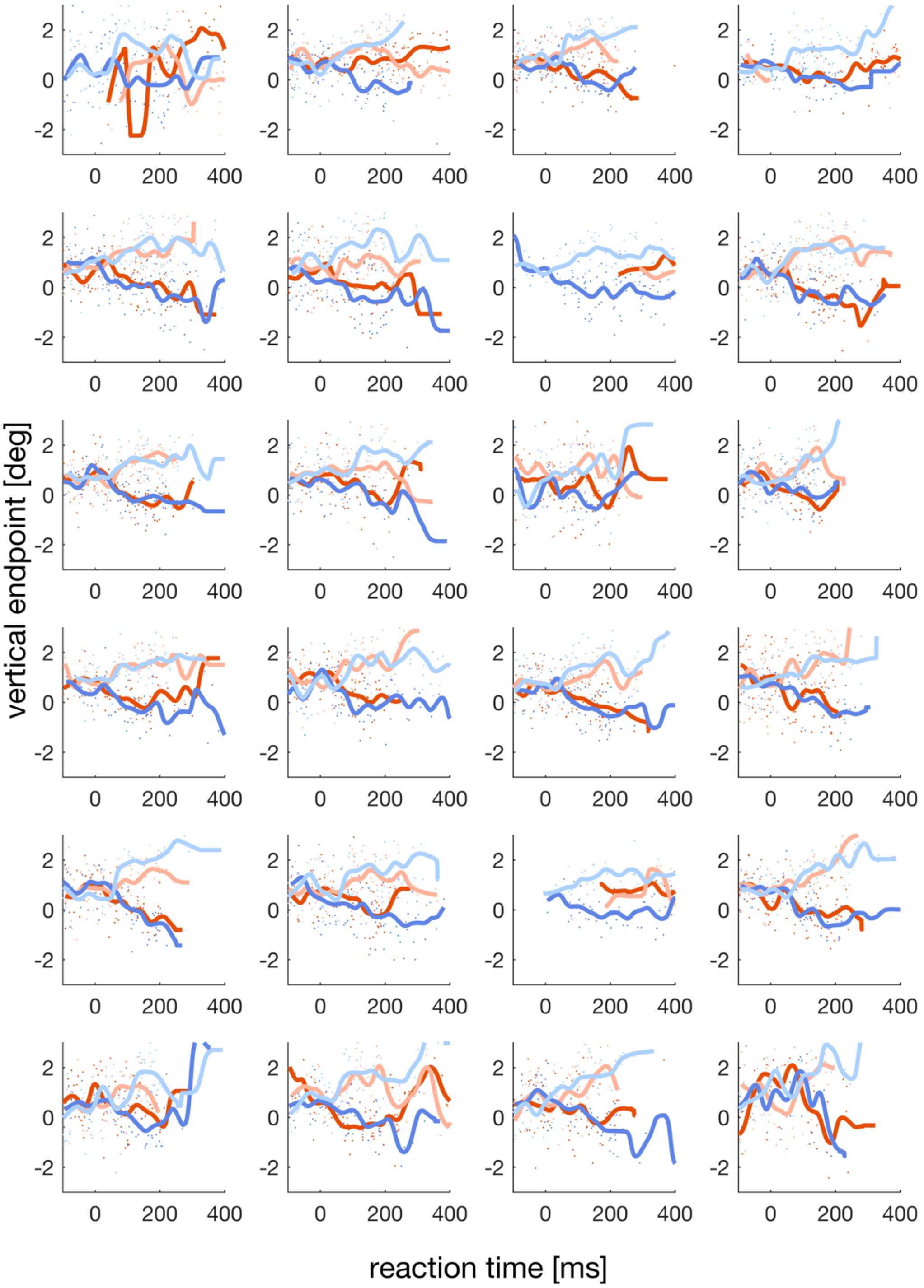

Suppl. Fig. S3. **Individual data Experiment 3 (cued onset)**. Vertical endpoints as a function of reaction time relative to onset of the vertical luminance bar for rewarded (orange) and unrewarded trials (blue). Saturated colors represent unflipped trials, faint colors flipped trials. Every panel denotes one participant, every data point denotes an individual trial. Lines are weighted averages, smoothed by a Gaussian window with a standard deviation of 16 ms.

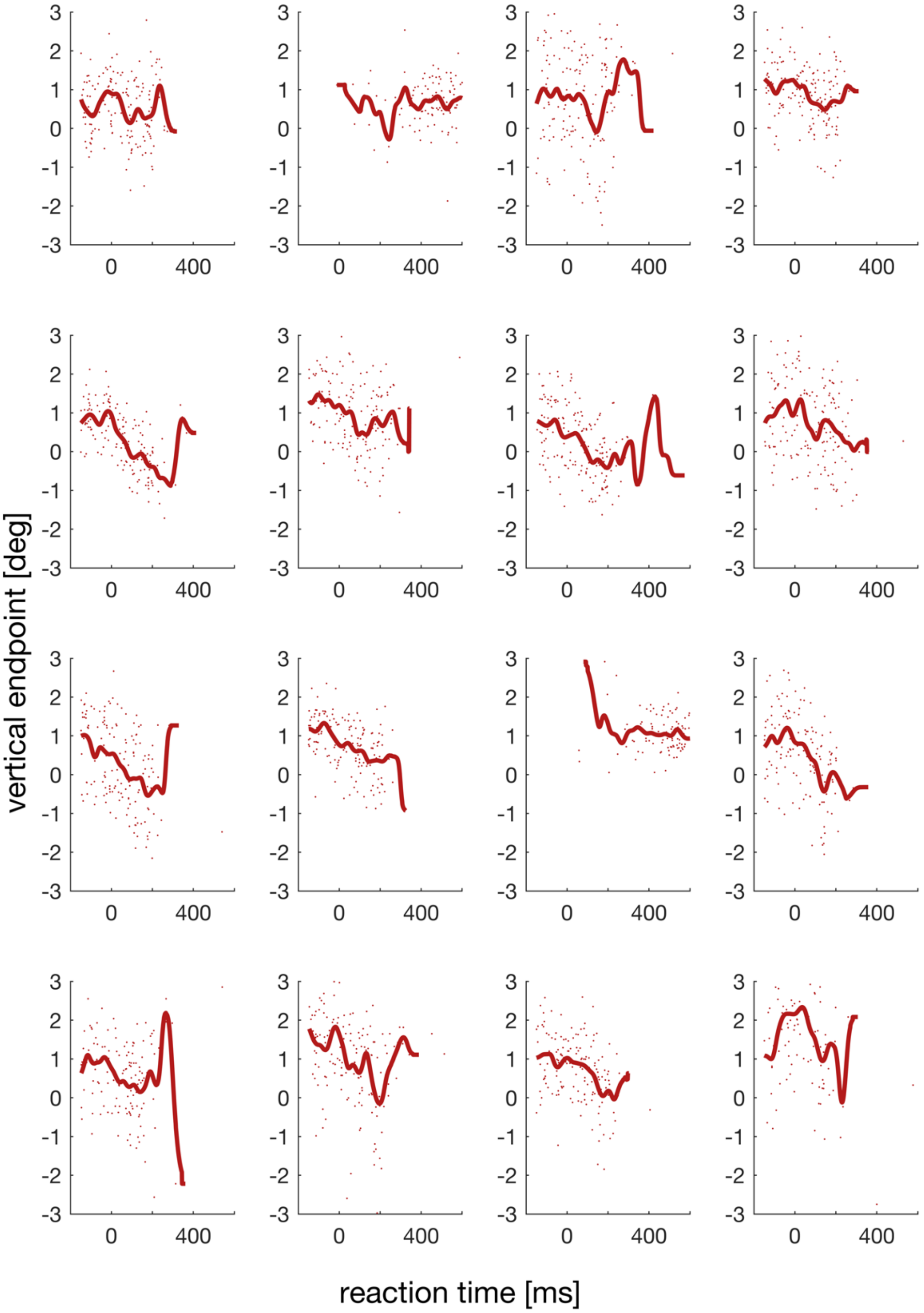

Suppl. Fig. S4. **Individual data Experiment 4 (predictable onset)**. Vertical endpoints as a function of reaction time relative to onset of the vertical luminance bar. Every panel denotes one participant, every data point denotes an individual trial. Lines are weighted averages, smoothed by a Gaussian window with a standard deviation of 16 ms.

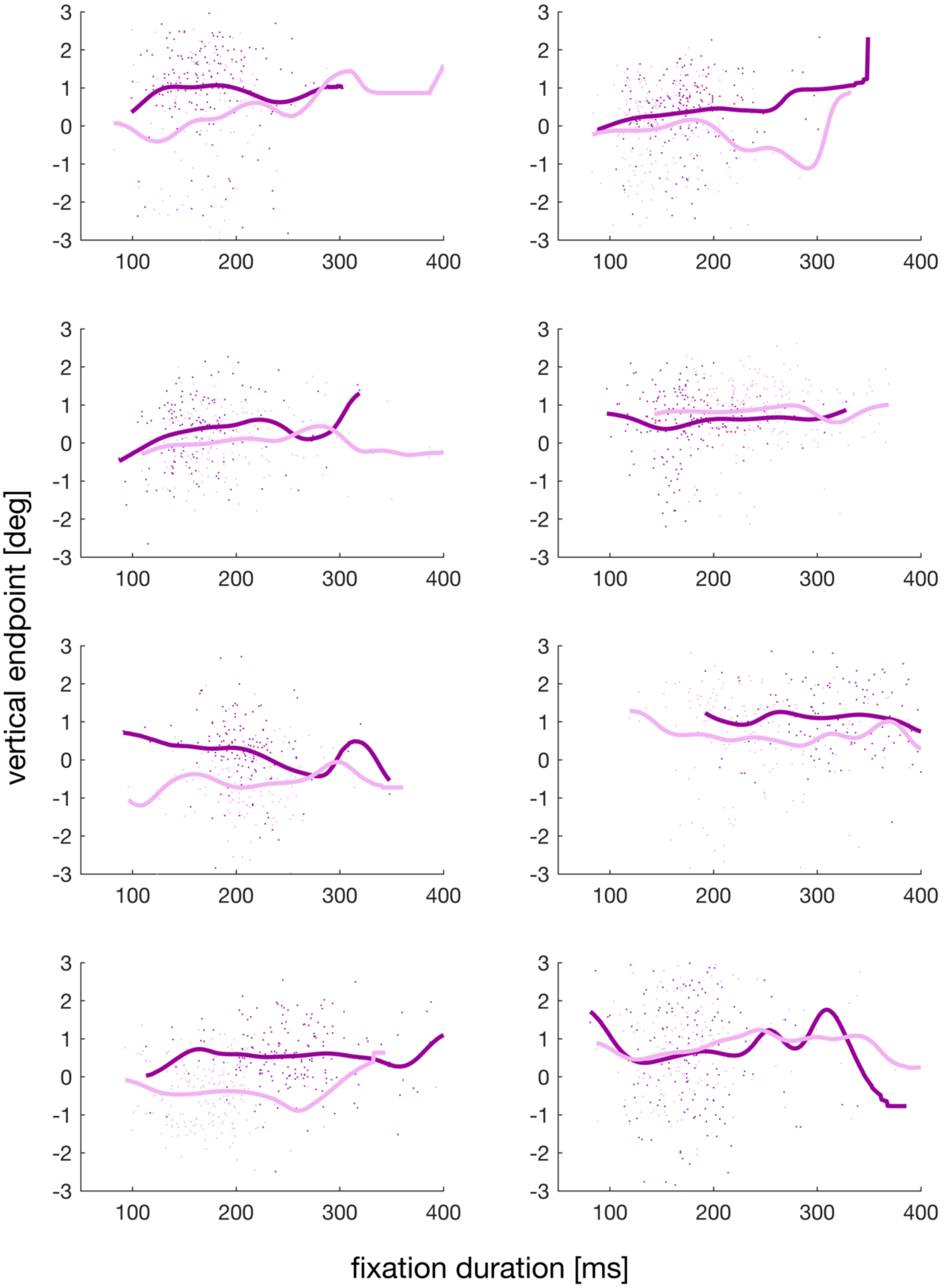

Suppl. Fig. S5. **Individual data Experiment 5 (preview quality)**. Vertical endpoints as a function of fixation duration when preview quality was good (saturated) or poor (faint). Every panel denotes one participant, every data point denotes an individual trial. Lines are weighted averages, smoothed by a Gaussian window with a standard deviation of 16 ms.

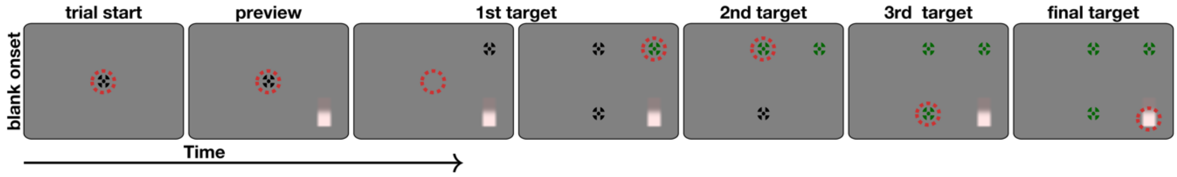

Suppl. Fig. S6. **Trial procedure Experiment 6 (blank onset)**. Participants started every trial by fixating a central fixation cross and pressing the space bar on a keyboard (first panel). The luminance bar appeared at its final location, either in the left or in the right hemifield (second panel). The initial central fixation cross and luminance bar were presented together for a total duration of 1.2 s and the luminance bar could be peripherally inspected during that time. In case participants shifted their gaze away from the central fixation cross during peripheral inspection, the luminance bar was removed until gaze went back to screen center. After the 1.2 s, the central fixation cross disappeared and the first saccade target, another fixation cross, appeared at the upper right (third panel). Once it had been fixated, it turned green and the remaining two saccade targets appeared (fourth panel). In blank trials, the luminance bar disappeared 100 ms after gaze arrived at the second saccade target (fifth panel). It re-appeared with an onset delay of 0, 100, 200, 300 or 400 ms after fixating the last fixation cross (sixth panel) and before the final response to the luminance bar was made (seventh and last panel). In trials without blank, the luminance bar was continuously displayed.

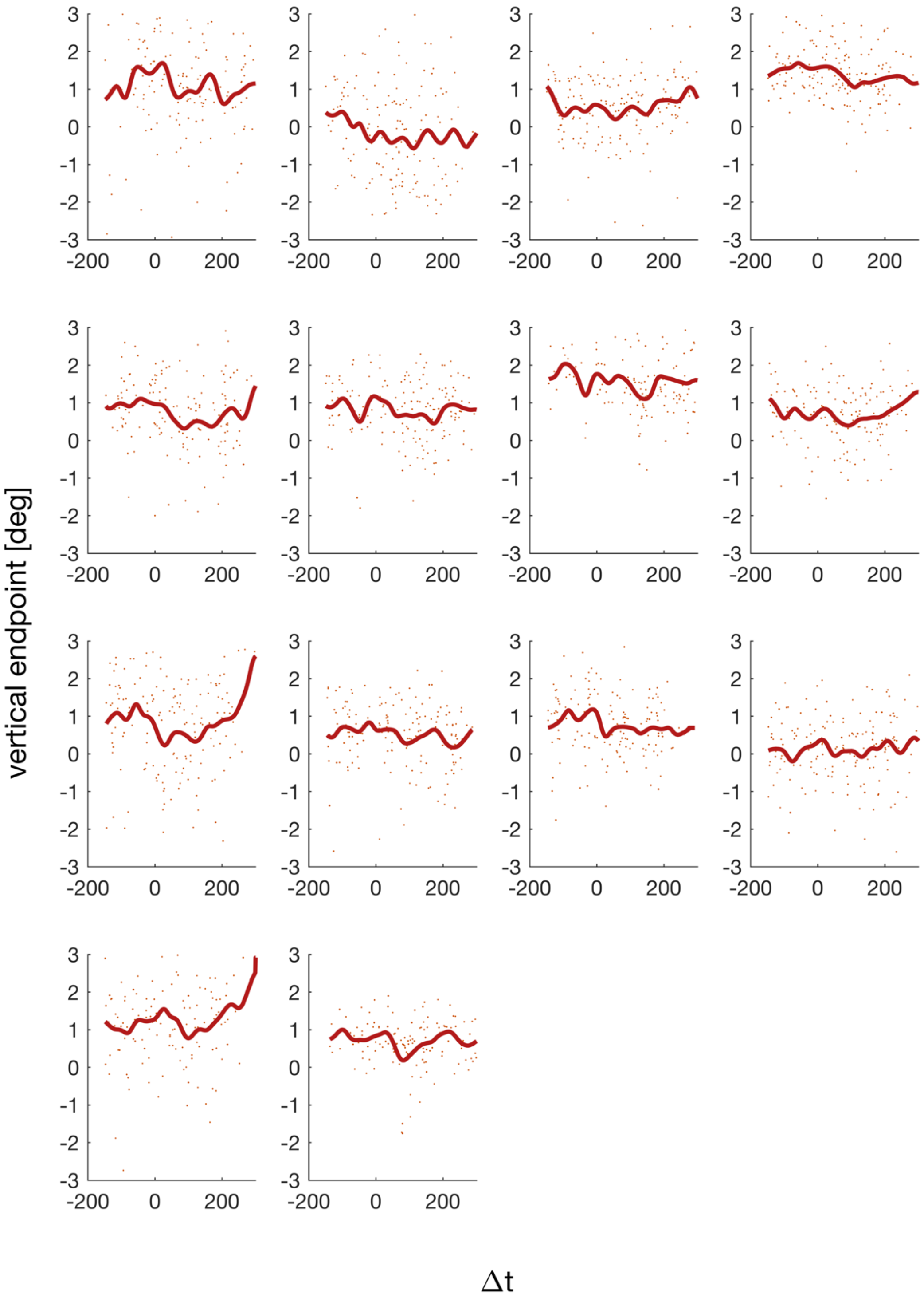

Suppl. Fig. S7. **Individual data Experiment 6 (blank onset)**. Vertical endpoints as a function of fixation duration when the target was blanked and re-appeared (t = 0) after fixating the last fixation cross. Every panel denotes one participant, every data point denotes an individual trial. Lines are weighted averages, smoothed by a Gaussian window with a standard deviation of 16 ms.
